# Potential Residual Pesticide Consumption: A Stratified Analysis of Brazilian Families

**DOI:** 10.3390/jox15020037

**Published:** 2025-03-01

**Authors:** Yan Lucas Leite, Tayna Sousa Duque, José Barbosa dos Santos, Elizângela Aparecida dos Santos

**Affiliations:** 1 Instituto de Ciências Agrárias, Universidade Federal dos Vales do Jequitinhonha e Mucuri, Unaí 38610-000, Brazil; yan.lucas@ufvjm.edu.br; 2 Departamento de Agronomia, Universidade Federal dos Vales do Jequitinhonha e Mucuri, Diamantina 39100-000, Brazil; tayna.duque@ufvjm.edu.br (T.S.D.); jbarbosa@ufvjm.edu.br (J.B.d.S.)

**Keywords:** agrochemical, dithiocarbamates, ethephon, food safety, law 14.785/2023, toxicology

## Abstract

Food safety is essential to ensure that food is safe for human consumption, particularly in light of the growing global and environmental changes, including population growth and climate variations. Meeting the increasing demand for food requires enhancing and protecting agricultural systems. A common strategy is the use of pesticides, which serve to protect cultivated plants from pests, diseases, and weeds. However, improper and excessive use of these products can lead to negative impacts, spanning economic, environmental, and human health aspects. Concerns about pesticide residues in food are global, as their effects on human health vary depending on exposure and quantity. The main objective of this study was to estimate the potential residual consumption (PRC) of pesticides present in food consumed by Brazilian households. Using a specific methodology, it was identified that pineapple had the highest average PRC (121.01 mg), primarily due to the high residue of the active ingredient ethephon. On the other hand, Dithiocarbamates showed the highest residual quantity. Tebuconazole was the most repeated in the samples. It was observed that the insecticide class was responsible for the highest average PRC in households, estimated at 142.45 mg annually, while higher-income families and those located in rural areas showed a greater propensity for potential residual pesticide consumption due to the higher consumption of fruits and vegetables. Additionally, it was found that households where the head of the family is male, highly educated, and older than 40 years present a higher risk of potential residual pesticide consumption. These results highlight the need for public policies focused on sanitary inspection, the training of professionals in the field, the rational use of pesticides by producers, and proper hygiene practices by consumers to mitigate health risks.

## 1. Introduction

Food security is defined as the physical and economic access to sufficient, safe, and nutritious food that meets the dietary needs and preferences for an active and healthy life [1]. This concept encompasses a range of measures and practices aimed at safeguarding consumers’ health, preventing foodborne illnesses, and ensuring the quality and integrity of food products, in addition to covering several interdisciplinary areas of knowledge [1,2].

Population growth and consumption patterns place a significant responsibility on food systems to provide goods continuously and safely to the population. By 2050, the global population is projected to approach 10 billion, increasing the demand for food by approximately 70% [3,4]. The growth in food demand is expected to be unevenly distributed across regions, with developing countries facing a more pronounced increase [5]. Furthermore, climate change has the potential to exacerbate food insecurity as it is closely linked to agricultural productivity [6]. In this context, it is crucial to intensify and protect agricultural systems to meet the growing food demand [3], with one of the strategies being the use of pesticides. The initial use behind these products was to combat hunger by increasing food production [7].

Currently, approximately 2.5 million tons of pesticides are used annually worldwide. In Brazil, pesticide consumption has surpassed 300,000 tons, reflecting a 700% increase over the past 40 years, making the country the largest consumer of pesticides and one of the largest producers of food commodities [7,8]. However, the incorrect and indiscriminate use of pesticides can have detrimental impacts on the environment, economy, and human health.

Pesticide residues in food are a global concern due to their potential impacts on human health, which depend on both the environment and the level of exposure [9,10]. As such, it is essential to monitor the residual levels present in foods consumed by the population, along with the socioeconomic characteristics associated with consumption. Families with higher income levels may be more exposed to pesticide residues due to their higher consumption of plant-based foods. Conversely, families in regions more exposed to extreme climatic conditions may experience higher levels of pesticide residue consumption due to the recurrent use of pesticides in agricultural production [11].

Several studies have examined residual exposure to pesticides, with research ranging from local to national levels [12,13,14,15]. In the United States, for instance, Yang et al. [14] analyzed 44 pesticides in 31 different food items, detecting high residue levels, such as the insecticide acetamiprid in green peppers. In terms of socioeconomic stratification, Donley et al. [16] reported that families in vulnerable situations are more exposed to the risks associated with pesticide residues, particularly due to residing in areas where intensive agricultural pesticide use is common. Moreover, individuals with higher income or education levels tend to adopt healthier diets, which paradoxically may increase indirect pesticide residue consumption due to a higher intake of fruits and vegetables [17].

According to Donley et al. [16], the main factors that increase pesticide residue exposure include geographical proximity to intensive agricultural areas, which raises the risk of air, soil, and water contamination due to pesticide drift during application. Additionally, socioeconomic inequalities result in low-income groups and racial minorities living in regions with higher pesticide usage, with limited access to mitigation and protective resources. Agricultural workers are among the most exposed, as they handle and apply pesticides directly, often without adequate protective equipment or sufficient training. This exposure is exacerbated by insufficient regulations and inadequate enforcement, contributing to excessive or improper pesticide use.

In Brazil, most studies are focused on the local level. Fraga G.P. [15], for example, assessed strawberry cultivation in Rio Grande do Sul and observed that 60% of the tested samples exceeded the maximum residue limit (MRL), with procymidone, carbendazim, and difenoconazole being the most frequently detected pesticides. Regarding individual characteristics, Gonzaga A.M. [18] investigated pesticide exposure and potential intoxication among the population of Mato Grosso, reporting that between 2001 and 2004, 84.1% of the 358 registered cases were men aged 19 to 45. Faria et al. [19] showed that in the Serra Gaúcha region, 86% of the 1479 agricultural workers exposed to pesticides were men, compared to 68% among women. Moura et al. [20] also identified higher exposure rates among men in the Alagoas region.

The likelihood of residual pesticide consumption increases significantly with the education level of the household reference individual [21]. Furthermore, exposure tends to be higher in households with older individuals due to a greater demand for foods considered healthy, such as fruits and vegetables [22,23,24].

In this research, potential residual consumption (PRC) is understood as the estimated quantity of pesticide residues that may be present in food following application. These residues can persist for varying durations and may be ingested or absorbed by humans or other organisms. The term “potential consumption” refers to the likelihood of exposure to these residues through the ingestion or use of contaminated products. This study aimed to estimate the potential consumption of pesticide residues in foods consumed by families in Brazil. Specifically, it examined the consumption of foods with the highest levels of pesticide residues; analyzed consumption patterns across different income levels and regions (Brazilian macro-regions and rural–urban areas); and identified consumer groups most exposed to residual ingestion based on gender, education, and age.

Thus, the present study stands out from previous research by conducting a stratified national-level analysis of potential residual pesticide consumption, overcoming the limitations of earlier studies that focused on local contexts, as suggested by Ferreira et al. [25] and Marques J.M.G. et al. [2]. The results obtained will contribute to mitigating the adverse health effects on consumers while providing a comprehensive analysis of the risks associated with potential foodborne intoxication and exposure, taking into account regional differences and the characteristics of Brazilian households. Furthermore, the study of indirect pesticide consumption by Brazilian households, stratified by various variables, will contribute to the scientific literature, as this approach remains underexplored in recent socioeconomic contexts. The findings may support public policies aimed at monitoring and promoting adequate consumption, encompassing aspects related to food production, supply, and consumption.

## 2. Materials and Methods

The estimation of potential residual pesticide consumption was conducted by adapting the methodology proposed by the World Health Organization [26], the Food and Agriculture Organization of the United Nations [27], and Ferreira et al. [25]. Initially, the potential residual pesticide consumption was estimated as follows:

(1) $${PRC}_{ij}=\sum_{k=1}^{m}R_{ik}*C_{kj}$$

where ${PRC}_{ij}$ is the potential residual consumption of the active ingredient i by family j, $R_{ik}$ is the residual concentration of i present in food k, and $C_{kj}$ is the quantity consumed of food k by family j.

To illustrate the calculation of the PRC of pesticides, let us consider the active ingredient ethephon, commonly used in pineapple cultivation. Assume that the residue concentration of ethephon in pineapple is 1.02 mg/kg and that family j consumes, on average, 118 kg of pineapple per year. Applying the previously presented formula, the estimated PRC for this family would be 120 mg of ethephon residue consumed annually (1.02 mg/kg × 118 kg). This calculation was repeated for each active ingredient found in the samples, allowing for a detailed estimation of the potential exposure to pesticide residues consumed by families throughout the year.

The data on pesticide concentration in food were obtained based on the results of the Pesticide Residue Analysis Program (PARA) by the National Health Surveillance Agency (ANVISA) for the years 2017/2018. PARA is a national initiative designed to monitor the presence of pesticide residues in food sold in the country. This program operates under rigorous scientific and regulatory standards, addressing various methodological stages to ensure the accuracy and representativeness of the results obtained. The first stage of PARA involves the meticulous selection of the foods to be evaluated, encompassing a wide range of food products that make up the basic diet of the Brazilian population. This selection considers variables such as seasonality, geographic origin, and cultivation methods to ensure sample representativeness. Subsequently, samples of these foods are collected in different regions of Brazil, following specific sampling protocols to ensure randomness and sample representativeness. The collected samples are then subjected to standardized preparation procedures, aiming at homogenization and minimization of interferences that may compromise the results of subsequent analyses [28].

The analysis itself is conducted in accredited laboratories, where samples are subjected to nationally and internationally recognized and validated analytical methods for the identification and quantification of pesticide residues. These analyses are performed using sensitive and specific techniques, ensuring detection even at minimal concentrations. The results of the analyses are then interpreted in light of the maximum residue limits established by Brazilian legislation, which determine the acceptable levels of pesticide residues in food. Interpreting these results is crucial for assessing the conformity of food with established safety standards and for supporting control and oversight actions. The results of the analyses are disseminated by ANVISA in technical reports and informative notes, providing relevant information to the population regarding the safety of food consumed in Brazil and guiding risk management measures and public health policies [28].

During the 2017–2018 analysis cycle, data provided by the ANVISA Pesticide Residue Monitoring Programme demonstrated a careful and representative sampling process. A total of 4616 samples were analyzed, collected from 14 plant-based foods that are representative of the Brazilian population’s diet, such as pineapple, lettuce, rice, and sweet potato, among others. These samples were acquired from retail establishments in 77 Brazilian municipalities, except the state of Paraná, which opted not to participate in the program from 2016 onwards. This geographically comprehensive approach aims to ensure that the sample reflects the diversity of foods consumed across Brazil. Furthermore, up to 270 different pesticides were investigated, providing a thorough analysis of the residues found. The results indicated that 77% of the samples were within the limits established by ANVISA for pesticide residues, with 49% of the samples showing no detected residues and 28% having levels equal to or lower than the maximum residue limit (MRL). The samples were collected between August 2017 and June 2018.

Therefore, the selection of foods and the collection of samples were carried out in a representative manner, to reflect consumption variations across the country. The methodology seeks to ensure transparency, addressing potential biases and providing a solid foundation for public policy decisions related to food safety and pesticide residue monitoring.

**Figure 1:**
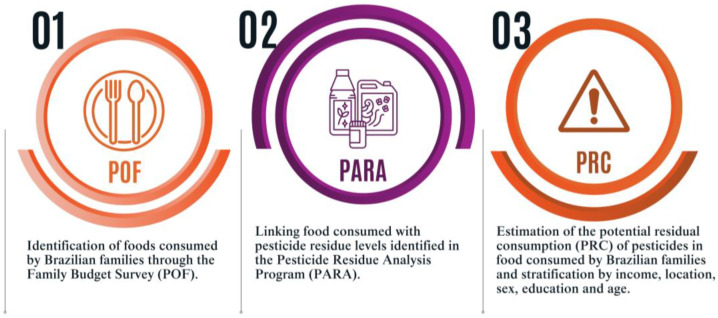
Methodological summary. Source: Self elaboration.

The data regarding the consumption patterns of Brazilian families and their socioeconomic characteristics were obtained from the information released by the 2017–2018 Family Budget Survey (POF). The POF is conducted by the Brazilian Institute of Geography and Statistics (IBGE) and aims to measure consumption structures, expenditures, and income of families, as well as the perception of living conditions of the population, according to household and individual characteristics. Being a sample survey, it provides socioeconomic data on the conditions of Brazilian families based on their household budgets [29]. Food consumption by families was grouped by different levels of income, education, gender, location, and age group. Data collection for the 2017–2018 POF took place between 11 July 2017, and 9 July 2018. The final sample contains 57,920 consumption units, corresponding to 69,017,704 families and 207,103,790 people (Figure 1).

Regarding ethical considerations, both the ANVISA Pesticide Residue Monitoring Programme (PARA) and the Brazilian Household Budget Survey (POF) data are sourced from official channels and adhere to stringent ethical protocols. PARA follows legal regulations for data collection and processing, ensuring that the samples are handled in an aggregated manner without identifying individuals, in line with data protection and food safety guidelines. ANVISA adopts practices in compliance with personal data protection regulations as stipulated by the General Data Protection Law (LGPD), and the data collected through the program are utilized for public policies related to food safety and pesticide residue control.

As for the IBGE’s POF, it is conducted under the principles set forth by the IBGE Ethics Committee, respecting the guidelines of the General Data Protection Law (LGPD). The data processing undertaken by POF complies with Article 23, Section I of Law No. 13.709/2018 (LGPD), ensuring transparency, purpose, and the procedures for personal data processing, guaranteeing that all collected data are used in an aggregated and anonymized manner, without identifying individual respondents. The legal framework for processing POF data is established in specific regulations, with the objective of analyzing consumption habits and the socioeconomic behavior of the Brazilian population, without violating individuals’ rights. The procedures ensure that data collection, storage, and processing are conducted securely, based on the need to fulfill statistical and public policy purposes. Ethical information is detailed in Ordinance No. 202, available at https://www.in.gov.br/en/web/dou/-/portaria-n-202-de-30-de-marco-de-2022-389908719 (accessed on 22 June 2023.). Further information on the purpose and procedures of data processing can be found on the official IBGE portal at https://www.ibge.gov.br/acesso-informacao/tratamento-de-dados-pessoais.html (accessed on 22 June 2023.).

Both data sets comply with local data protection regulations and have been utilized in accordance with relevant ethical and legal standards, ensuring the privacy and security of personal data.

## 3. Results

### 3.1. Estimation of Food Consumption and Pesticide Residual Levels

**Figure 2:**
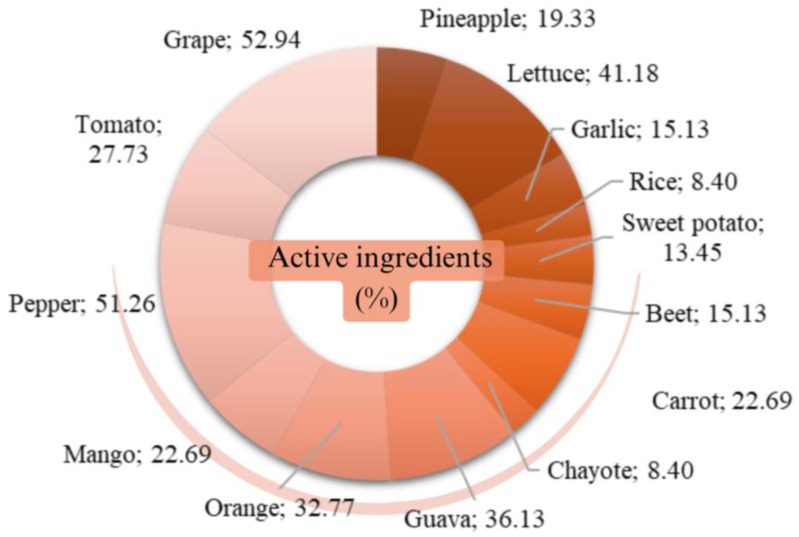
Percentage of active ingredients found in unsatisfactory samples (%). Primary data obtained from PARA [28].

The percentage of active ingredients in 14 foods in unsatisfactory samples was analyzed, i.e., irregularly detected, such as prohibited use in the country, not authorized for the crop, and a result value higher than the maximum residue limit (MRL) allowed for the analyzed crop [28] (Figure 2).

In total, 119 types of residual active ingredients were found, with grapes being the food with the highest number (63 different active ingredients), followed by bell pepper (61 different active ingredients). Conversely, the foods with the lowest number of residual active ingredients were rice (10 different active ingredients) and chayote (10 different active ingredients). Additionally, the most frequent active ingredient was tebuconazole.

Brazilian families consume, on average, 270.33 kg of rice per year, being the most consumed food; on the other hand, garlic is the least consumed food, with an average annual consumption of 16.70 kg. Regarding active ingredients, dithiocarbamates are present in larger quantities in lettuce (1.75 mg), beetroot (0.49 mg), and mango (0.21 mg). The highest potential residual consumption was estimated for ethephon in the pineapple sample (121.02 mg/year), and the lowest value for acephate identified in carrot (04.57 mg/year) (Table 1).

**Figure 3:**
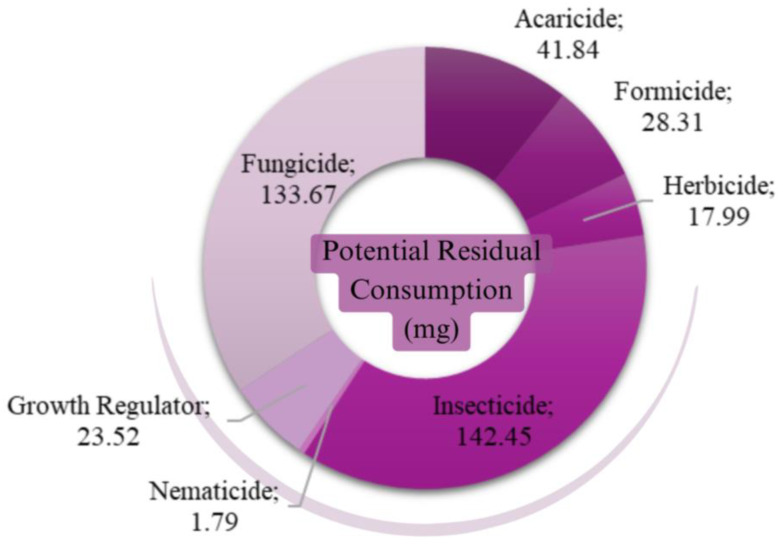
Estimate of annual average potential residual consumption (PRC) per pesticide class per household (mg). Source: Primary data obtained from POF [29] and PARA [28]. In Table A1 of Appendix A, there is a summary of the statistical description of the specified variables.

The PRC values were estimated for the 119 types of active ingredients identified in the 14 types of food. After that, the PRC values for the 119 types of pesticides were grouped into different classes (Figure 3). (The active ingredients were classified according to the AGROFIT MAPA classification, available at https://agrofit.agricultura.gov.br/agrofit_cons/principal_agrofit_cons. Accessed on 22 June 2023.)

In the estimation of potential residual consumption, it is observed that the insecticide class stands out (142.45 mg/year) and then fungicides with 133.67 mg/year. On the other hand, the nematicides class registers a considerably lower value of 1.79 mg/year (Figure 3).

### 3.2. Estimation of Food Consumption at Different Income Levels and Locations

**Figure 4:**
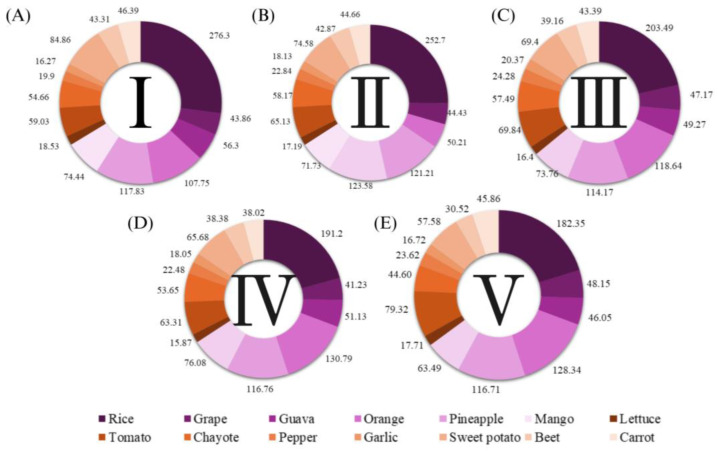
Average household consumption in kg year^−1^ according to annual per capita income class in Brazilian reais. Class I: Less than BRL 20,000.00 (**A**); class II: from BRL 20,000.00–BRL 40,000.00 (**B**); class III: from BRL 40,000.00–BRL 60,000.00 (**C**); class IV: from BRL 60,000.00–BRL 80,000.00 (**D**); class V: above BRL 80,000.00 (**E**). Income in January 2018 values. The statistical results of the MANOVA (Multivariate Analysis of Variance) test showed that there are statistically significant differences between income groups regarding the consumption of different foods. *F*-values were 25.53 (Wilks), 25.38 (Pillai), 25.68 (Lawley), and 74.80 (Roy). The *F*-value indicates the ratio between the variance explained by the model and the residual variance, with Prob > *F* = 0.0000. The very low *p*-value (<0.001) indicates that the differences between income strata are statistically significant. Source: Primary data obtained from POF [29] and PARA [28].

Figure 4 presents the average food consumption by household annual income strata, classified into five categories, expressed in kg per year.

Households in class I have a higher consumption of rice, guava, lettuce, sweet potato, beetroot, and carrot compared to other classes. On the other hand, in class V, there is a higher average consumption of grapes and tomatoes (Figure 4).

**Figure 5:**
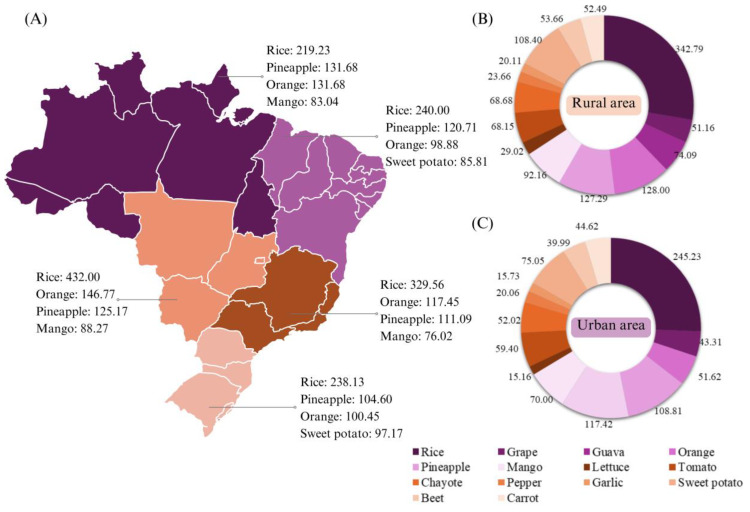
Average household food consumption by Brazilian macro-region (**A**), and in rural (**B**) and urban (**C**) areas in kg year^−1^. The results indicate that the variable “location” has a statistically significant impact on the quantities of food consumed by households. For all variables, the *p*-values were below 0.05, suggesting that the differences in consumption between households are not attributable to chance, but rather to a real effect of the household’s location. Furthermore, the high *F*-statistics (>10) demonstrate that the model used significantly explains the variation in the quantities consumed, reinforcing the robustness of the observed differences between households in different contexts. The *p-* and *F*-values were obtained using the ANOVA test. Source: Primary data obtained from POF [29].

Regarding the location of Brazilian households, Figure 5 illustrates consumption patterns across different macro-regions—north, northeast, southeast, central-west, and south—as well as in urban and rural areas.

### 3.3. Estimation and Identification of Consumers Most Exposed to Residual Ingestion

Table 2 below presents the potential residual pesticide consumption, disaggregated by pesticide class and stratified by income level and location.

A clear pattern of increasing PRC is observed as one moves from lower to higher income classes. This trend is consistent across all pesticide categories analysed. *p*-values below 0.0001 for all categories indicate that the differences between income classes are statistically significant, while the high *F*-values, such as 163.46 for acaricides and 143.04 for insecticides, confirm the robustness of these differences. Regarding household location, PRC exhibits less pronounced variations between urban and rural areas compared to income classes. For most pesticides, the estimated PRC in rural areas is slightly higher, particularly for insecticides and fungicides. The *p*-values associated with the tests for location-based differences show that, for acaricides (*p* = 0.3870), there is no statistically significant difference, whereas for the other categories, the differences are significant, reflecting distinct consumption patterns between urban and rural zones. Concerning macro-regions, the results indicate considerable variation in PRC. The Central-West region recorded the highest PRC values for most pesticides, whereas the North exhibited the lowest levels. All *p*-values are below 0.0001, indicating statistically significant differences between regions, with high *F*-values, such as 203.85 for insecticides and 142.07 for fungicides, highlighting a strong effect of geographical location on PRC.

Additionally, Table 3 provides the estimated PRC for each pesticide class, taking into account the individual characteristics of the household reference person, including sex, education level, and age.

Regarding sex, the mean PRC is slightly higher for men across all pesticide classes, except for nematicides and growth regulators, where differences are minimal. The greatest discrepancies are observed for acaricides (43.15 mg for men vs. 39.97 mg for women) and fungicides (138.90 mg for men vs. 126.23 mg for women). Furthermore, *p*-values below 0.0001 for nearly all categories indicate statistically significant differences between men and women, except for growth regulators (*p* = 0.7520). The high *F*-values, particularly for fungicides (*F* = 38.28) and insecticides (*F* = 30.19), further support the robustness of these differences. Concerning education, the PRC systematically increases with educational attainment, reaching the highest values among individuals with a completed higher education degree, such as 184.27 mg for insecticides and 163.72 mg for herbicides. Conversely, individuals with no formal education exhibit the lowest PRC values. All *p*-values are below 0.0001, demonstrating statistically significant differences between groups, while the high *F*-values, such as 64.1 for acaricides and 56.94 for insecticides, confirm the influence of education on PRC variation. The age-based analysis reveals a progressive increase in PRC as age advances, with the highest values recorded among individuals aged 40 years and older. In contrast, younger age groups, particularly those up to 20 years, present the lowest values. This pattern may be associated with differences in dietary and consumption habits over the life course. All *p*-values are below 0.0001, indicating statistically significant differences between age groups, while the high *F*-values reinforce the influence of age on PRC.

The findings highlight significant variations in the potential residual consumption (PRC) of pesticides across different socioeconomic and demographic factors, reinforcing the importance of considering these aspects in risk assessments and policy interventions. The presence of 119 types of active ingredients in food samples, with grapes and bell peppers exhibiting the highest diversity, underscores the complexity of pesticide exposure in the Brazilian diet. The predominance of insecticides and fungicides in PRC estimates suggests their substantial contribution to overall pesticide exposure. Socioeconomic disparities are evident as PRC increases with income, education level, and age, indicating differentiated consumption patterns that may reflect access to diverse food sources and dietary preferences. Geographical factors also play a crucial role, with the central-west region recording the highest PRC levels and the north the lowest, highlighting regional disparities in pesticide exposure. While urban–rural differences are less pronounced, rural households tend to exhibit slightly higher PRC levels, particularly for insecticides and fungicides. Gender-based variations, though moderate, indicate higher PRC among men. These results emphasize the necessity for targeted policies addressing pesticide regulation, food safety, and exposure mitigation, considering the multifaceted nature of pesticide consumption patterns across Brazilian households.

## 4. Discussion

Exposure to multiple pesticides through diet is a growing concern, as food often contains mixtures of different chemical compounds, whose cumulative and synergistic effects are still poorly understood [31]. Data from the Pesticide Residue Analysis Programme (PARA) reveal that 23% of the tested samples contained banned pesticides or concentrations exceeding permitted limits, with bell pepper, guava, and carrot standing out [28]. Furthermore, 0.89% of the analyzed samples indicated an acute health risk.

Agricultural pesticides are classified into six toxicological categories, ranging from extremely toxic (Category 1, red band) to unlikely to cause acute harm (Category 5, blue band), in addition to unclassified products (green band). Among the compounds frequently detected above permissible limits, tebuconazole, dithiocarbamates, and ethephon belong to Category 5, while acephate is classified as Category 4 [32]. Tebuconazole, a systemic triazole fungicide, is widely used in approximately 60 crops for controlling fungal diseases by inhibiting ergosterol biosynthesis [33,34,35]. Its persistence varies from 8 days in fruits to 40.8 days in soil, with detections in human biological fluids, indicating significant environmental exposure [34,36,37,38,39]. Studies associate tebuconazole with carcinogenic risks, endocrine disruption, and reproductive toxicity [40,41]. Dithiocarbamates, broad-spectrum and low-cost, are used in over 70 crops, inhibiting essential biochemical processes in phytopathogens [42,43,44]. Their degradation generates ethylene thiourea, a carcinogenic compound [45,46,47]. Propineb, a common dithiocarbamate, has maximum residue limits (MRLs) ranging from 0.05 to 50.0 mg/kg [48]. Ethephon, a plant growth regulator, is used to stimulate ripening and flowering, as well as functioning as a pesticide [49,50,51,52]. Ethephon residues are associated with renal, hepatic, and neurological risks, in addition to reproductive dysfunctions and adverse effects on pregnant women [53,54,55]. Bell pepper and grape samples frequently exceed the MRL for this compound [56,57].

Insecticides, essential for pest control, include organophosphates, carbamates, pyrethroids, and neonicotinoids, which are associated with genotoxic, neurotoxic, and reproductive effects [58]. Studies report increased risks of neurological diseases in agricultural workers with chronic exposure [59,60]. The higher exposure to pesticide residues in plant-based foods and fruits in certain regions of Brazil or among higher-income groups can be explained by various factors. In regions with large-scale agricultural production, such as the central-west and south of Brazil, intensive pesticide use is more common due to the need to increase productivity, especially in monocultures. These intensive agricultural practices result in frequent pesticide use to ensure high yields and maintain product appearance, highlighting the need for stricter regulations.

In this regard, Brazil has made progress with the approval of Law No. 14,785 of 27 December 2023, which established new guidelines for the control, inspection, and supervision of pesticides in the country. Originally, the bill proposed centralizing these responsibilities exclusively within the Ministry of Agriculture and Livestock (Mapa), removing competencies from the Brazilian Institute of Environment and Renewable Natural Resources (Ibama) and the National Health Surveillance Agency (Anvisa). However, following presidential vetoes, the tripartite evaluation model was preserved, whereby Mapa, Ibama, and Anvisa share responsibilities in assessing the impacts of pesticides on human and animal health and the environment. The new legislation sets deadlines for registration analysis, authorizes temporary registrations for research, and maintains restrictions on the reuse of packaging [61].

In terms of the stratification of our findings, the discussions indicate that older groups, predominantly men with higher education levels, also have a higher consumption of fresh fruits and vegetables, which may contain more pesticide residues, especially if not organic. Access to imported products, which often have higher pesticide residue levels due to differing regulatory standards in producing countries, also contributes to this exposure. In some regions, enforcement of pesticide use regulations may be insufficient, exacerbating the presence of residues in food reaching consumers. Additionally, management practices in some large-scale agricultural areas, including the continuous use of pesticides to combat pests and diseases, further increase the presence of these residues. Thus, higher exposure to pesticide residues is often associated with higher consumption patterns and the agricultural practices in producing regions, particularly in areas with intensive farming and less stringent pesticide use controls.

Furthermore, dietary consumption in Brazil is dominated by rice, coffee, beans, bread, and beef [62], reflecting regional variations in expenditure and associated differences in pesticide exposure levels [63]. Records of poisoning are more frequent in regions with intensive pesticide use [64,65]. Socioeconomically vulnerable individuals may also be more exposed to risks, with evidence of significant exposure among children and minority populations [16,66,67].

Food and nutritional security is, therefore, strongly impacted by the presence of pesticide residues, highlighting the need for rigorous monitoring, effective regulation, and strategies to reduce exposure, especially in vulnerable populations. The limitations of this study include the influence of seasonal variations and regional regulations on pesticide residues, as well as potential inaccuracies in self-reported dietary data. Additionally, the analysis of the cumulative and synergistic effects of chronic pesticide ingestion remains a challenge, given the complexity of interactions between different chemical compounds and their long-term impacts on human health. Gradual and continuous exposure to low concentrations of pesticides may not be immediately perceptible but is associated with adverse chronic effects, reinforcing the need for preventive policies. Although Brazilian pesticide legislation has evolved in recent years (Law 14,785/2023), gaps still require improvement. Thus, strengthening public policies is recommended through the implementation of advanced monitoring technologies, the promotion of educational programs, fostering collaboration across sectors, and adapting regulatory frameworks to keep pace with scientific and technological advancements in the field.

## 5. Final Considerations

The present study provides a detailed overview of the potential consumption of pesticide residues in foods consumed by Brazilian households, highlighting important correlations with socioeconomic and geographical variables. The data indicate that the consumption of foods containing pesticide residues is significant, with relevant implications for public health. Foods such as grapes, bell peppers, and pineapples exhibit high levels of residues, including toxic compounds such as tebuconazole, dithiocarbamates, and ethephon.

The analysis of different income brackets reveals that households with higher purchasing power tend to consume more plant-based foods, increasing their exposure to pesticide residues. Similarly, the geographical distribution of consumption indicates that rural areas and specific macro-regions of Brazil exhibit distinct consumption patterns, contributing to unequal exposure to residues.

These findings suggest that it is essential to strengthen public food security policies, with an emphasis on the monitoring and control of pesticide residues in food. Additionally, there is an urgent need to promote sustainable agricultural practices that minimize pesticide use, as well as to raise awareness of the risks associated with the consumption of contaminated foods.

With the projected increase in food demand due to population growth, effective strategies must be implemented to ensure that the food produced is safe and healthy, without compromising consumer health. The adoption of stricter regulations and the dissemination of good agricultural practices can have a significant impact on reducing residue levels in the food supply chain.

For future research, it is recommended to conduct more comprehensive studies that assess the cumulative and synergistic effects of pesticide residues, as well as the development of alternative pest control methods that are less harmful to the environment and human health. It is also important to investigate innovative education and awareness strategies for consumers and producers to promote healthier and more sustainable food choices across all segments of the population.

## Figures and Tables

**Table 1:** Food commodities (Commodities), household consumption (kg year^−1^) (HC), active ingredient with highest residual level (IA), code FRAC (FRAC), group name (Group), quantity of active ingredient (mg kg^−1^) (Quantify), and estimate of potential residual consumption (PRC) (mg).

Commodities	HC	AI	FRAC	Group	Quantity	PRC
Pineapple	118.65 ^(63.40)^	Ethephon	-	-	1.02	121.02
Pineapple	18.10 ^(95.98)^	Dithiocarbamates	M 03	Dithiocarbamates and relatives (Electrophiles)	1.78	32.21
Garlic	16.70 ^(97.48)^	Imazalil	3	DMI-fungicides (DeMethylation Inhibitors)	1.1	18.37
Rice	270.33 ^(107.16)^	Carbendazim *	1	MBC-fungicides (Methyl Benzimidazole Carbamates)	0.08	21.62
Sweet potato	80.99 ^(78.21)^	Carbendazim *	1	MBC-fungicides (Methyl Benzimidazole Carbamates)	0.21	17
Beet	42.54 ^(69.30)^	Dithiocarbamates	M 03	Dithiocarbamates and relatives (Electrophiles)	0.49	20.84
Carrot	45.77 ^(72.32)^	Acephate	-	-	0.1	4.57
Chayote	55.06 ^(74.48)^	Acephate	-	-	0.26	14.31
Guava	54.87 ^(87.93)^	Famoxadone	11	QoI-fungicides (Quinone outside Inhibitors)	0.23	12.62
Orange	111.85 ^(92.06)^	Pyrimethanil	9	AP-fungicides (Anilino-Pyrimidines)	0.38	44.5
Mango	73.48 ^(85.45)^	Dithiocarbamates	M 03	Dithiocarbamates and relatives (Electrophiles)	0.21	15.43
Pepper	20.62 ^(84.86)^	Thiacloprid	-	-	0.52	10.72
Tomato	60.96 ^(81.18)^	Dichlorvos	-	-	0.36	21.94
Grape	44.29 ^(71.59)^	Cypermethrin	-	-	0.51	22.58

Note: * Carbendazim (Thiophanate + Carbendazim). The values in superscript refer to the coefficient of variation (cv). The consumption of the foods considered the annual average per household in kg. The active ingredients were obtained by averaging the values in the unsatisfactory samples available in PARA. Specifically, this table considered the active ingredients with the highest residual values present in each food. The PRC values were obtained according to Equation (1) described in Section 2. Source: Primary data obtained from POF [29] and PARA [28]. Pesticides were classified according to FRAC [30]. In Table A1 of Appendix A, there is a summary of the statistical description of the specified variables.

**Table 2:** Potential residual consumption (PRC) (mg) of pesticide by income class, rural or urban locations, and macro-region.

	Acaricide	Formicide	Herbicide	Insecticide	Nematicide	Growth Regulator	Fungicide
Income class							
I	38.05	26.49	16.96	130.99	1.70	21.41	125.21
II	54.14	32.82	20.34	179.42	2.06	28.84	162.35
III	59.34	35.26	22.94	196.67	2.10	30.68	170.96
IV	62.39	35.38	22.62	198.57	2.15	28.59	175.15
V	66.65	40.30	25.16	221.61	2.36	34.96	187.45
*p*-values	0.0000	0.0000	0.0000	0.0000	0.0000	0.0000	0.0000
*F*-values	163.46	63.77	51.29	143.04	40.63	13.46	90.73
Locations							
Rural	41.32	31.91	19.22	150.68	1.88	17.87	147.71
Urban	41.99	27.44	17.68	140.11	1.78	24.80	129.67
*p*-values	0.3870	0.0000	0.0000	0.0000	0.0052	0.0000	0.0000
*F*-values	0.75	69.03	19.21	18.56	7.82	27.29	62.8
Macro-region							
North	24.19	21.39	13.22	85.59	1.41	22.77	91.07
Northeast	40.03	27.13	18.15	131.19	1.72	29.37	124.43
Southeast	47.16	28.62	18.96	157.22	1.90	18.69	145.71
Midwest	51.50	32.55	19.98	182.69	2.10	22.70	170.46
South	46.84	31.27	17.79	168.45	1.76	19.37	148.79
*p*-values	0.0000	0.0000	0.0000	0.0000	0.0000	0.0000	0.0000
*F*-values	162.86	49.05	39.93	203.85	38.29	20.29	142.07

Note: The PRC values were obtained according to Equation (1) described in Section 2. Source: Primary data obtained from POF [29] and PARA [28]. The *p-* and *F*-values were obtained using the ANOVA test.

**Table 3:** Potential residual consumption (PRC) (mg) of pesticide by sex, education and age of the reference person in the household.

	Acaricide	Formicide	Herbicide	Insecticide	Nematicide	Growth Regulator	Fungicide
Sex							
Man	43.15	29.29	18.49	147.39	1.85	23.47	138.90
Woman	39.97	26.95	17.29	135.43	1.71	23.61	126.23
*p*-values	0.0000	0.0001	0.0006	0.0000	0.0001	0.7520	0.0000
*F*-values	23.03	16.06	11.67	30.19	14.58	0.1	38.28
Education							
Without instruction	32.04	22.96	15.51	109.87	1.49	18.65	112.14
Incomplete Primary	39.14	27.53	17.42	136.45	1.75	19.44	130.83
Complete Primary	41.79	28.32	17.61	142.37	1.79	25.27	133.99
Incomplete Secondary	36.67	24.68	16.40	123.29	1.62	20.14	116.60
Complete Secondary	42.75	28.04	17.81	144.08	1.79	25.75	132.60
Incomplete Higher	43.50	27.10	17.13	144.71	1.74	26.88	131.92
Complete Higher	55.92	34.42	21.78	184.27	2.09	30.28	163.72
*p*-values	0.0000	0.0000	0.0000	0.0000	0.0000	0.0000	0.0000
*F*-values	64.1	32.11	24.6	56.94	18.57	11.24	32.78
Age							
Up to 20 years	21.45	20.72	13.81	77.60	1.16	21.89	84.16
20 to 40	35.36	25.14	16.43	120.51	1.60	19.85	114.14
40 to 60	43.21	29.18	18.41	148.22	1.83	25.31	138.05
60 to 80	45.95	29.92	18.80	155.36	1.92	24.24	146.36
Above 80 years	46.55	28.92	18.38	151.53	1.89	21.81	141.40
*p*-values	0.0000	0.0000	0.0000	0.0000	0.0000	0.0001	0.0000
*F*-values	80.99	39.37	22.23	89.83	35.25	9.11	84.94

Note: The PRC values were obtained according to Equation 1 described in Section 2. Source: Primary data obtained from POF [29] and PARA [28]. The p and *F* values were obtained using the ANOVA test.

## Data Availability

The original contributions presented in this study are included in the article. Further inquiries can be directed to the corresponding author(s).

## Appendix A

**Table A1:** Summary statistics of the main variables.

Variable	Observation	Mean	Standard Deviation	Minimum	Maximum
Rice (kg per year)	13,009.00	270.33	289.71	0.21	3120.00
Grape (kg per year)	2353.00	44.29	31.71	1.46	728.00
Guava (kg per year)	1201.00	54.87	48.25	0.68	606.01
Orange (kg per year)	5646.00	111.85	102.98	1.51	1489.75
Pineapple (kg per year)	2009.00	118.65	75.22	18.46	733.20
Mango (kg per year)	2158.00	73.48	62.79	2.60	888.89
Lettuce (kg per year)	6125.00	18.10	17.37	0.21	218.40
Tomato (kg per year)	11,239.00	60.96	49.49	1.92	942.08
Chayote (kg per year)	1770.00	55.06	41.01	0.21	520.00
Pepper (kg per year)	3720.00	20.62	17.50	0.52	218.56
Garlic (kg per year)	5073.00	16.70	16.28	0.10	169.52
Sweet potato (kg per year)	2707.00	80.99	63.34	1.66	780.00
Beet (kg per year)	1414.00	42.54	29.48	0.16	430.72
Carrot (kg per year)	4981.00	45.77	33.10	1.51	832.16
PRC_Acaricide (mg per year)	27,764.00	41.84	53.19	0.00	960.96
PRC_Formicide (mg per year)	20,271.00	28.31	30.39	0.29	693.02
PRC_Herbicide (mg per year)	21,759.00	17.99	20.74	0.05	652.23
PRC_Insecticide (mg per year)	27,764.00	142.46	170.14	0.02	2772.93
PRC_Nematicide (mg per year)	18,572.00	1.79	1.93	0.01	34.06
PRC_Growth Regulator (mg per year)	11,706.00	23.53	55.60	0.01	762.50
PRC_Fungicide (mg per year)	27,764.00	133.68	157.93	0.05	2375.82
Per capita income (annual)	56,585.00	14,545.50	23,382.34	8.41	1,001,420.00
Urban (%)	56,585.00	0.77	0.42	0.00	1.00
North (%)	56,585.00	0.14	0.35	0.00	1.00
Northeast (%)	56,585.00	0.33	0.47	0.00	1.00
South (%)	56,585.00	0.14	0.35	0.00	1.00
Southeast (%)	56,585.00	0.26	0.44	0.00	1.00
Midwest (%)	56,585.00	0.12	0.33	0.00	1.00
Age (years)	56,585.00	49.85	15.77	12.00	107.00
Without instruction (%)	56,585.00	0.09	0.29	0.00	1.00
Incomplete Primary (%)	56,585.00	0.40	0.49	0.00	1.00
Complete Primary (%)	56,585.00	0.08	0.28	0.00	1.00
Incomplete Secondary (%)	56,585.00	0.05	0.22	0.00	1.00
Complete Secondary (%)	56,585.00	0.23	0.42	0.00	1.00
Incomplete Higher (%)	56,585.00	0.03	0.17	0.00	1.00
Complete Higher (%)	56,585.00	0.12	0.32	0.00	1.00
Woman (%)	56,585.00	0.41	0.49	0.00	1.00

Note: The PRC values were obtained according to Equation (1) described in Section 2. Source: Primary data obtained from POF [29] and PARA [28].
